# School of cheminformatics in Latin America

**DOI:** 10.1186/s13321-023-00758-0

**Published:** 2023-09-19

**Authors:** Karla Gonzalez-Ponce, Carolina Horta Andrade, Fiona Hunter, Johannes Kirchmair, Karina Martinez-Mayorga, José L. Medina-Franco, Matthias Rarey, Alexander Tropsha, Alexandre Varnek, Barbara Zdrazil

**Affiliations:** 1 Institute of Chemistry, Campus Merida, National Autonomous University of Mexico, Merida‑Tetiz Highway, Km. 4.5, Ucu, Yucatan Mexico; 2 LabMol – Laboratory for Molecular Modeling and Drug Design, Faculdade de Farmacia, Universidade Federal de Goias, Goiania, GO Brazil; 3 European Molecular Biology Laboratory, European Bioinformatics Institute (EMBL-EBI), Wellcome Genome Campus, Hinxton, CB10 1SD Cambridgeshire UK; 4 Division of Pharmaceutical Chemistry, Department of Pharmaceutical Sciences, University of Vienna, Josef-Holaubek-Platz 2, 2D 303, 1090 Vienna, Austria; 5 Institute for Applied Mathematics and Systems, Merida Research Unit, National Autonomous University of Mexico, Sierra Papacal, Merida, Yucatan Mexico; 6 DIFACQUIM Research Group, Department of Pharmacy, School of Chemistry, National Autonomous University of Mexico, Avenida Universidad 3000, 04510 Mexico City, Mexico; 7 ZBH - Center for Bioinformatics, Universität Hamburg, Bundesstraße 43, 20146 Hamburg, Germany; 8 Molecular Modeling Laboratory, UNC Eshelman School of Pharmacy, University of North Carolina at Chapel Hill, Chapel Hill, NC 27599 USA; 9 Laboratoire d’Infochimie, UMR 7177 CNRS, Université de Strasbourg, 4, Rue B. Pascal, 67000 Strasbourg, France

**Keywords:** Exploratory data analysis, Chemography, On-demand compound catalogs, Natural products, ChEMBL, Zika, Computational toxicology, Latin America, Cheminformatics

## Abstract

We report the major highlights of the School of Cheminformatics in Latin America, Mexico City, November 24–25, 2022. Six lectures, one workshop, and one roundtable with four editors were presented during an online public event with speakers from academia, big pharma, and public research institutions. One thousand one hundred eighty-one students and academics from seventy-nine countries registered for the meeting. As part of the meeting, advances in enumeration and visualization of chemical space, applications in natural product-based drug discovery, drug discovery for neglected diseases, toxicity prediction, and general guidelines for data analysis were discussed. Experts from ChEMBL presented a workshop on how to use the resources of this major compounds database used in cheminformatics. The school also included a round table with editors of cheminformatics journals. The full program of the meeting and the recordings of the sessions are publicly available at https://www.youtube.com/@SchoolChemInfLA/featured.

## Introduction

The primary purpose of the school was to enhance the cheminformatics field. It was targeted but not limited to the community in Latin America. This school, organized by a Latin American country, was held in Mexico City on November 24th and 25th, 2022. The virtual meeting featured talks by 10 international experts. Table 1 summarizes the full program. The set of speakers covered a broad perspective as they work in academia, big pharma, and public research institutions. One thousand one hundred eighty-one participants registered from seventy-nine different countries, including Mexico, India, Brazil, Colombia, United States, Pakistan, and Peru. They included 715 from America (678 from Latin America, which represents 57% of the total number of registrants), 277 from Asia, 92 from Africa, and 97 from Europe. The group of participants consisted of 52% of undergraduate and graduate students, 27% professionals, 12% postdoctoral researchers, and 9% with other non-disclosed profiles.

**Table 1:** Program of the School of Cheminformatics in Latin America

	Speaker, *Affiliation,* Country	Title
Day 1	Alexandre Varnek *University of Strasbourg,* France	Chemography concept in chemical space analysis
	Matthias Rarey *University of Hamburg,* Germany	Beyond screening: cheminformatics for billion-sized make-on-demand compound catalogs
	Johannes Kirchmair *University of Vienna,* Austria	Cheminformatics in natural product-based drug discovery
	Round table: Rajarshi Guha Karina Martinez Mayorga José Luis Medina Franco Matthias Rarey Alexander Tropsha Barbara Zdrazil Austria–Germany–Mexico–USA	Topics: * “Dos and don’ts” when writing a paper * Where to get help/orientation to publish your work
Day 2	Barbara Zdrazil & Fiona Hunter *EMBL-EBI,* UK	Workshop: ChEMBL—accessing big molecular data via the web interface and API
	Carolina Horta Andrade *University of Goias,* Brazil	Cheminformatics-driven discovery of hits for neglected and emerging diseases
	Rajarshi Guha *Vertex Pharmaceuticals,* USA	Integration then interrogation: exploratory data analysis on a deadline
	Alexander Tropsha *University of North Carolina,* USA	Methods and models for chemical toxicity prediction

The talks were accessible through Zoom and YouTube. Available recordings of talks and the full program are freely available at https://www.youtube.com/@SchoolChemInfLA/featured. The following sections summarize the key developments presented and discussed during the meeting. The content is organized into seven presentations plus a round table.

## Integration then interrogation: exploratory data analysis on a deadline; speaker Rajarshi Guha

Exploratory data analysis (EDA) is a well-studied topic in statistics that helps to get better analytics. A valuable aspect of EDA is to generate questions about the data beyond the general original ones. Once initial questions are answered, it becomes evident that additional data is needed to unveil new questions that would have been undetected otherwise. EDA involves assessing the distributions of the data, looking for correlations, performing dimension reduction, and exploring different representations. EDA is domain-specific, so initial assessments include analysis of whether the data follows a distribution and the identification of biases. Key strategies of EDA related to chemical data include assessing the distributions, looking for correlations, performing dimension transformations, and exploring different representations. EDA is essential for many scientific tasks, such as predictive model development, image analysis, and high-throughput screening (HTS).

In HTS, a large amount of data is produced about molecule's activities (e.g., percentage of inhibition) and there might be secondary data or a secondary assay run for a subset. In other cases, the data is compared to public sources like ChEMBL [1], which contains available data on other assays. Since the data can be generated or gathered from different sources, the normality, dependence and linearity of the data are relevant for the assessment of i suitability for predictive modeling, and for the selection of the type of model to generate. Image analysis is also used: in a high throughput phenotypic screen, the morphology of cells is normally analyzed, looking at correlations between the readouts and statistics on individual channels, as there also might be artifacts to consider.

EDA deals with the entire dataset; splitting into training and test sets is not performed. It also has inherent biases, such as artifacts and correlations, that need to be acknowledged and considered. EDA is used to identify clusters of compounds in physicochemical property spaces. Predictive models are used to assess the suitability of the data, while image analysis is used to reduce noise.

Thus, there are important constraints we operate under that need to be acknowledged:

- Data can be big or small.
- Data is updated or missing.
- There are many different data types; they need to be identified and integrated
- Integration is driven by the types of questions we want to answer.
- Some data types may be regulated.
- Usually we need to respond quickly (days to weeks).

These constraints imply that the appropriate infrastructure needs to be in place to enable integration and that EDA needs to be efficient, well-executed, and communicated to the scientific team. An important aspect of efficient communication is the way the data is presented and visualized. To draw conclusions from the data, tools, and workflows must be used to manipulate it, and the analysis must be transmitted to collaborators.

## Chemography concept in chemical space analysis; speaker Alexandre Varnek

Chemography is a tool for “big data” analysis, with approximately 10^9^ compounds physically available, up to 10^26^ structures stored in proprietary databases, and 10^33^ compounds with drug-like properties that could be synthesized. Oprea [2] proposed the chemography approach to build maps describing the structure and properties of molecules using cheminformatics techniques. Generative topographic mapping (GTM) is a method that transforms the initial space of *n* descriptors into a two-dimensional space.

### GTM concepts

The GTM algorithm transforms the initial space of *n* descriptors into a two-dimensional space called latent space by introducing a flexible sheet-shape function called a manifold. In the initial *n*-dimensional space, the data is modeled by an ensemble of Gaussian functions localized at the nodes of a rectangular grid superimposed on the manifold. In the latent space, the projection of a given molecule is described by the probability of its location in different nodes of the rectangular grid.

The transformation of the ensemble of the probabilities, called responsibilities, gives place to a vector whose length is equal to the number of nodes. The vectors can be understood as descriptors of each molecule. When the molecules are added, the projection of the vectors results in a density landscape, which shows the density distribution of the molecules in the chemical space. This density landscape is very convenient for analyzing regions that are well-populated or underpopulated and the distribution of different chemotypes. There are three types of GTM landscapes: a *density landscape* characterizing data density distribution, a *class landscape* characterizing population of activity classes (active or inactive) and an *activity or property landscape* which reports an average activity or property values in a given part of the map.

### Technical details

GTM maps can be built with different descriptors, such as ISIDA fragment descriptors [3, 4], which can vary by topology, size, and atom labels. Two scenarios can be considered to profile a chemical library with respect to different activities: preparation of single-task models for particular activities or construction of one universal map able to predict the entire pharmacological profile in a multitasking manner. A prototype of the universal map was constructed for the ChEMBL database containing about 1.7 million compounds, which delineates the biological relevant chemical space and is able to predict > 700 biological activities. This gave rise to the ChemSpace Atlas [5] tool which contains approximately 1.5 billion compounds, > 40,000 hierarchically related maps and > 1.5 million activity landscapes. GTM applications include virtual screening, analysis of large chemical collections, AI-driven design of new molecules and reactions, and cartography and AI-driven design of new molecules.

## Beyond screening: cheminformatics for billion-sized make-on-demand compound catalogs; speaker Matthias Rarey

About 20 years ago most people were doing virtual screening on existing compound collections. In 1998 Lewell et al. [6] tried to create new molecules by recombining fragments. They described a set of cutting rules and used them to shred molecules into pieces and to recombine the parts to form new compounds. At the same time a reduced graph descriptor was developed [7] where the molecule is also cut it into smaller pieces and every piece is described by a set of nodes. In 2001 both findings were put together and a special search method was developed [8], which starts with a query and instead of looking at one molecule after another, selects molecules from the chemistry space of small fragments. Moving forward, an interesting transition happened [9] when researchers were looking to create small fragments by mapping potential small molecules as reactants for the reaction and describe all the plausible chemical reactions available in the library. So, this method gives them much higher reliability on the synthetic accessibility of compounds.

Recently, in 2019 Marcus Gastreich [10] summarized advances in modeling chemical space classification and their recent growth rates. Basically, all those databases were created by using reaction rules and certain fragments. Pharmaceutical companies extended this concept even further by taking more and more potential reactants and additional reactions to make these spaces much larger. The large number of molecules and possible reactions makes the enumeration of molecules from fragments almost infeasible and will consume a lot of time to process. So, several methods have been published to solve this by combining fragment spaces and topological descriptors [11, 12].

An approach to overcome the limitation of methods based on topology is through the optimization via metaheuristics which can be combined with arbitrary scoring functions. In 2022, Meyenburg et al. [13] presented Galileo, a novel genetic algorithm to sample fragment spaces which in combination with a novel pharmacophore mapping approach, called Phariety, enables 3D searches in fragment spaces. The 3D pharmacophore models have proven to be particularly useful as filters for virtual screening. Nowadays we see a lot of interest in larger collections which are then not available physically, but in make-on-demand catalogs which are provided by many companies. Already, for a decade we have seen interest in constructing these kinds of spaces.

Exploration of chemical space [14] remains in the core of research among the chemical informatics community. Following a different direction, make-on-demand catalogs describe chemistry in an integrated way without the enumeration of all the molecules. Such catalogs are orders of magnitude larger, and they substantially impact the early phase of drug discovery, even for challenging 3D searches [15].

## Cheminformatics in natural product-based drug discovery; speaker Johannes Kirchmair

Natural product drug discovery is challenging due to limited availability and high costs of materials, difficulties in harvesting, transporting, isolating, testing, and resynthesis, and problems related to decomposition, aggregation, precipitation, and chemical reactivity. Computational and cheminformatics methods offer in silico approaches, but 3D in silico approaches depend on correct stereochemistry. Many computational methods have been devised from and designed for organic synthetic drug-like molecules rather than natural products, and to use them also in the natural product space, some modifications and adaptations may be required.

Chen and Kirchmair [16] summarized the state-of-the-art, scope, and limitations of computational methods in natural-products-based drug discovery. They covered six major areas of application: data curation, analysis, visualization, navigation, and comparison of the chemical space; quantification of natural product-likeness; prediction of bioactivities; ADME and safety profiles; natural-products-inspired de novo design; and prediction of natural products prone to interfere with biological assays.

Natural products databases such as the SuperNatural 3.0 [17] database offer big data downloads, and the BIOFACQUIM database [18], developed at the National Autonomous University of Mexico (UNAM) which collects natural products isolated and characterized in Mexico offers big downloads and virtual screening. Several other natural product databases in the public domain have been reviewed [16].

A machine-learning approach, reported in 2019 [19], identifies natural products with high accuracy and can be used to quantify drug-likeness in large molecular databases. The method classifies small molecules as natural products or synthetic molecules using similarity maps that highlight important atoms. It can also quantify the natural-product-likeness of small molecules and identify natural products in large molecular databases.

## Workshop. ChEMBL—accessing big molecular data via the web interface and API; led by Barbara Zdrazil and Fiona Hunter

The first launch of the ChEMBL database (www.ebi.ac.uk/chembl) in 2009 [1] was a milestone in the recent history of chemical biology and drug discovery because it provided unprecedented free access to large amounts of high-quality, curated data on bioactive molecules. ChEMBL has grown significantly since then and now impacts a wide range of areas that include drug discovery, data science, and the development and validation of AI, machine learning, and other in silico methods.

The ChEMBL database links drug-like compounds to their biological targets via experimental bioactivity data. UniChem is a database that produces cross-references between chemical structure identifiers from different databases (www.ebi.ac.uk/unichem). Both ChEMBL and UniChem can be accessed through an interactive web interface as well as programmatically via web services.

A general introduction to ChEMBL and UniChem was presented, and the following topics were discussed:

(i) What is ChEMBL and how is it structured?
(ii) What types of data does ChEMBL contain?
(iii) The process to extract and curate bioactivity data
(iv) Sources of drug data for ChEMBL, and its curation and integration
(v) What is UniChem?
(vi) A demonstration of ChEMBL web interface
(vii) Other methods to access ChEMBL data (via download, sematic web, or API)
(viii) Worked examples to access ChEMBL programmatically using the API endpoints.

Further information is available via free training resources:

- ChEMBL webinar: https://www.ebi.ac.uk/training/events/guide-explore-drug-compounds-and-their-biological-targets-using-chembl/.
- ChEMBL quick tour: https://www.ebi.ac.uk/training-beta/online/courses/chembl-quick-tour/.
- ChEMBL & UniChem API webinar: https://www.ebi.ac.uk/training/events/guide-accessing-chembl-and-unichem-through-api/.

For questions about using ChEMBL or UniChem, the user can check the documentation and Frequently Asked Questions (www.ebi.ac.uk/chembl), or email at chembl-help@ebi.ac.uk or unichem@ebi.ac.uk for queries, feedback, and suggestions.

## Cheminformatics-driven discovery of hits for neglected and emerging diseases; speaker Carolina Horta Andrade

The Zika virus, reported for the first time in 1947, is biologically fascinating; sadly, the associated disease is being neglected, for drug discovery applications [20] and has now spread to more than 50 countries. To address this issue, the Open Zika project [21] was launched. It is a global collaboration with the goal of accelerating the discovery of an effective treatment for the infection. The project enabled the performance of massive docking-based virtual screening campaigns for all Zika virus proteins and the development of machine-learning models to predict the cytoprotective effect of compounds over Zika virus infection. As a result of the combined computational and screening efforts, several compounds were identified: five no-nucleoside compounds that inhibit the Zika virus polymerase, one dual inhibitor of the virus protease and polymerase, and eight compounds that were able to protect the glioblastoma cells from Zika virus infections.

The research group then turned to the COVID-AI project [22, 23] which focuses on the discovery of anti-COVID-19 agents. The workflow consisted of nine steps: in silico assays, target-based assays, cytotoxicity assays, cell-based SARS-CoV-2 assays, chemical synthesis, in vitro ADMET, in vivo DMPK, development of drug-targeted nanoparticles, and in vivo SARS-CoV-2 models. The best model for cytopathic effect assays was developed using available information from PubChem, MACCS key descriptors, and the random forest machine learning algorithm. For the main protease, a virtual screening campaign of compound databases was performed. The first compounds obtained showed medium-to-high potency.

## Methods and models for chemical toxicity prediction; speaker Alexander Tropsha

A pivot-point in modern chemical toxicity modeling was the conception of Tox21, in 2007. Tox21 aims to move chemical toxicity to a mechanistic explanation and away from animal testing. This has been pursued by developing tools and protocols that rely on in vitro data and mechanistic modeling of toxicity. Historically, testing of chemicals has been performed on animal models. This practice requires the use and sacrifice of several animals and is occasionally valuable for building predictions to extrapolate to humans. These analyses have been done without a deep understanding of why chemicals cause certain types of toxicity. Several tests have been run on thousands of chemicals at EPA and FDA, as well as in universities and biotech companies. In addition, the strong statement from US EPA in 2019 to eliminate all mammal chemical testing for toxicity by the year of 2035, imposed pressure on the development of alternative toxicity testing methods.

There are two major historical trends: read across and statistical methods. “Read across” is a relatively simple chemical similarity-based method with the key aspect of extracting structural alerts or chemical toxicity alerts [23]. Chemical fragments are presumed to be associated with a specific type of chemical toxicity. QSAR (statistical) models are directly computed from chemical structures. Historically, regulatory agents have been given higher recognition to the chemical categories under the read-across method, mainly due to the transparency and mechanistic interpretability associated with this method.

The OECD QSAR Toolbox is an open-access tool used by regulators to fill in gaps in current knowledge. It is based on grouping chemicals into categories, presuming that in the read-across approach, a new chemical of interest has the particularly expected toxicity because of its similarity with other chemicals of the same chemical category. In QSAR methods, there is no mechanistic assumption. Instead, there is a hypothesis that one could build statistical models to correlate a chemical descriptor matrix with the target property and expect to get a strong correlation between the actual and the predicted biological activity or toxicity values.

Typically, the alerts are derived from a large collection of chemical structures that happen to have a particular chemical functional group. For example, in the work reported by Braga et al. [24] molecules were predicted by alerts as blockers but experimentally are nonblockers of hERG, while QSAR model predictions agreed with the experimental data.

Hybrid strategies combine building statistical QSAR models and interpreting them in terms of significant chemical fragments and using these models to assess the significance of structural alerts derived solely from looking at the structure of toxic compounds without any statistical modeling. In most cases, model accuracy and prediction can be improved by integrating chemical and biological descriptors, typically by concatenating them.

Chemical-biological read-across (CBRA) [25] is a methodology that learns from two sets of neighboring molecules: biological and chemical. It outperforms other models and exhibits consistently high external classification accuracy and applicability to diverse chemicals. Chemistry-wide association studies (CWAS) [3] explore how chemical structures are associated with activity by integrating statistical and non-statistical modeling. This model derives alerts from validated QSAR models and validates alert-based assertions by QSAR. In this way, the toxicity predictions are both interpretable and statistically significant.

The use of popular algorithms such as AI and deep learning has gained more attention in recent years. The statistical accuracy of deep-learning-based models seems to have been very high. However, potential problems should be taken into account, for example, verifying if the data used on the model was heterogeneous, adequate and curated. Importantly, the lack of data curation could represent a major weakness. When the data is not curated, for example, when there are duplicates, the model is expected to show over-optimistic accuracy. However, the accuracy of curated data is more realistic and shows the innate inaccuracy of the data but also shows an honest assessment of model accuracy.

When analyzing publications that use the most innovative methods, it is important to look at the data carefully and see if statements made are justified by the data. Models, even in the age of deep learning, need to be built with proper division of the data set into training and test set, and external validation. All the best practices need to be preserved.

In computational toxicology, the times are good and exciting, as the amount of data is large and growing, and the importance of building reliable models of computational toxicity prediction is increasing.

## Quotes from the round table with editors and beyond

Rajarshi Guha

When you apply methods, be sure you applied statistics properly.

If people think of deep learning as the next step beyond random forest for regression analysis, that is a dead end.

In the future, cheminformaticians will couple AI or ML with chemical structure generation to influence synthetic chemists in the design of new molecules.

Karina Martinez

Focus on the main topic, avoid unnecessary wordiness.

Learn to follow ethics guidelines.

Be moved by the aim of communicating your ideas and contributions, not by Journal Impact Factor or fashion.

Documentation gives transparency.

When building models, consider flexibility, temporality and nondeterminism.

Jose Medina

If you are not an expert, have a sense of the usefulness and the limitations so that you can interpret the outcome of programs you use.

Always read the documentation of the programs you use.

Preprints are becoming more popular; they are a good way to disseminate the work, and for making the work available to the community.

Matthias Rarey

There are two main types of research papers: application and development of methods.

Pay attention to the language, reach out to colleagues for proof reading, and especially ask English native speakers to peer review your work.

Alexander Tropsha

(About social media) Don´t self-glorify but distribute the knowledge anyway you can.

(About predatory journals) just ignore them, you cannot fight them, you cannot eliminate them, but you could ignore them, you should ignore them.

If your work focuses on applications, you must validate.

If you just use something from the web and make predictions about molecules it is worth almost nothing.

Barbara Zdrazil

Bring your message out in a simple and clear way.

Predictions can never be better than the underlying data (garbage in – garbage out).

By providing more and better-quality data in a fair way, we will enable better predictions in the future.

It is the practice of writing what gives you the skills.

Improve your writing skills by writing, reading, and reviewing papers.

Others

The structure of a language determines (or greatly influences) the modes of thought and behavior characteristics of the culture in which it is spoken – Sapir & Whorf.

The limits of the tool shape my reality – Bridget Cogley.

In drug discovery, the treasure is in the molecules – John Van Drie.

## Conclusions

The virtual School of Cheminformatics in Latin America, Mexico City, November 24, 25, 2022, gave an overview of recent developments of chemical space enumeration, applications of cheminformatics to natural products research with focus on drug discovery, drug discovery for neglected diseases, toxicity prediction, and data analysis. The school also featured a workshop on using of the broadly available resources from ChEMBL, focusing on how to use the resources of this major compounds database used in cheminformatics. The school included a round table to discuss topics related to scientific publishing. The event was part of a continued effort in Mexico to contribute to developing the rigorous practice of cheminformatics in Latin America [26] in an open format available to the scientific community worldwide that, in this school, was attended by participants from seventy-nine countries. It is anticipated that in the next few years, the Latin American community will be more integrated with cheminformatics and associated topics being developed worldwide. It is expected that the present school will be part of a continued and sustained effort to join other research and educational events on cheminformatics that have being happening for several years now such as the School of Chemoinformatics or Pharmacy Informatics held at the University of Strasbourg in France, or the University of Vienna in Austria. It is expected that future editions of this school will be hybrid in order to benefit from face-to-face discussions and to facilitate the rapid dissemination and contact with interested persons for whom traveling is a burden.

## Data Availability

Complete recordings of available sessions are freely available on YouTube here https://www.youtube.com/@SchoolChemInfLA/featured.
